# Controlled Deposition of Particles in Porous Media for Effective Aquifer Nanoremediation

**DOI:** 10.1038/s41598-017-13423-y

**Published:** 2017-10-11

**Authors:** Carlo Bianco, Janis Eneida Patiño Higuita, Tiziana Tosco, Alberto Tiraferri, Rajandrea Sethi

**Affiliations:** Department of Environment, Land and Infrastructure Engineering (DIATI), Politecnico di Torino, Corso Duca degli Abruzzi 24, 10129 Torino, Italy

## Abstract

In this study, a model assisted strategy is developed to control the distribution of colloids in porous media in the framework of nanoremediation, an innovative environmental nanotechnology aimed at reclaiming contaminated aquifers. This approach is exemplified by the delivery of humic acid-stabilized iron oxide nanoparticles (FeOx), a typical reagent for *in situ* immobilization of heavy metals. By tuned sequential injections of FeOx suspensions and of solutions containing a destabilizing agent (i.e. calcium or magnesium), the two fronts, which advance at different rates, overlap at the target location (i.e., the central portion) of the porous systems. Here, the particles deposit and accumulate irreversibly, creating a reactive zone. An analytical expression predicting the position of the clustering zone in 1D systems is derived from first principles of advective-dispersive transport. Through this equation, the sequence and duration of the injection of the different solutions in the medium is assessed. The model robustness is demonstrated by its successful application to various systems, comprising the use of different sands or immobilizing cations, both in 1D and 2D geometries. The method represents an advancement in the control of nanomaterial fate in the environment, and could enhance nanoremediation making it an effective alternative to more conventional techniques.

## Introduction

Nanoremediation is an innovative technology employed for the remediation of contaminated aquifers^1,2^. It involves subsurface injection of a reactive suspension of engineered nanoparticles for *in situ* degradation, transformation, or immobilization of pollutants^3–5^. Specifically, nanoparticles are injected directly into and/or in the proximity of the source of contamination to create a reactive zone for the treatment of the dissolved contaminant phase and the abatement of the polluting plume. Nanoremediation is a non-invasive and flexible approach, which can significantly reduce the time needed for the restoration of the site even in presence of strongly recalcitrant pollutants^2^.

The key feature of reactive nanomaterials applied to groundwater remediation is their high specific surface area, essential for the rapid degradation of a wide variety of recalcitrant contaminants. Relevant examples of reactive materials include: nanoscale zerovalent iron particles, mainly used against chlorinated solvents^3,6,7^; Carbo-Iron, a composite material combining zerovalent iron and activated carbon for the simultaneous adsorption and degradation of hazardous organic compounds^8,9^; and iron oxide (FeOx) nanoparticles for enhanced biodegradation of organics^10,11^ and irreversible immobilization of heavy metals^12,13^.

FeOx nanoparticles are commonly coated with a saturated layer of humic acids, which ensures colloidal stability even in suspensions containing large amounts of particles (in the order of 10 g/L)^14^. Transport tests previously conducted with these suspensions showed that particles are highly mobile under conditions typically found in subsurface environments^12^. In field applications, this could result in an undesirable distribution of the particles, which would continue their migration in the aquifer, bypassing the polluted areas and potentially representing a secondary source of contamination for humans and the environment. Indeed, questions have been raised about the safety of introducing nanoparticles into the subsurface^2,15,16^, because of their potential ecotoxicity^1,17,18^ and their capability to facilitate the transport of contaminants adsorbed on their surface^19–21^. Moreover, despite the enormous potential of this technology, its large-scale implementation is still hindered by limitations related to the injection strategy and by the cost of the nanomaterials^17^.

Therefore, the greatest challenges faced by engineers to advance nanoremediation are the effective delivery and the appropriate dosing of the nanoparticles into the subsoil^22,23^, necessary for the correct emplacement of the *in situ* reactive zone and to minimize costs. Nanoparticles need to be precisely delivered to the target zone and to be stable over time, to extend the lifetime of the remediation and to prevent their uncontrolled migration.

In this study, an innovative strategy is tested to control the distribution and immobilization of nanoparticles in porous media. The proposed approach consists in the sequential injection of a stable suspension of reactive nanoparticles and of a destabilizing agent, from the same injection point, with the aim of creating a reactive zone within a targeted portion of the contaminated aquifer where the two fronts overlap. An analytical transport expression guides the design of the injection sequence to achieve the desired particle distribution at a target location of the medium. The applicability and flexibility of the model and of the injection strategy is tested experimentally for the immobilization of FeOx nanoparticles in both 1D and 2D setups mimicking saturated subsoil. The robustness of the method is challenged by varying the chemistry and the granulometry of the media, and by using different destabilizing agents. The main potential advantages of this strategy in field applications are finally discussed.

## Results and Discussion

### Injection strategy achieves concentrated particle distribution at targeted location

**Figure 1:**
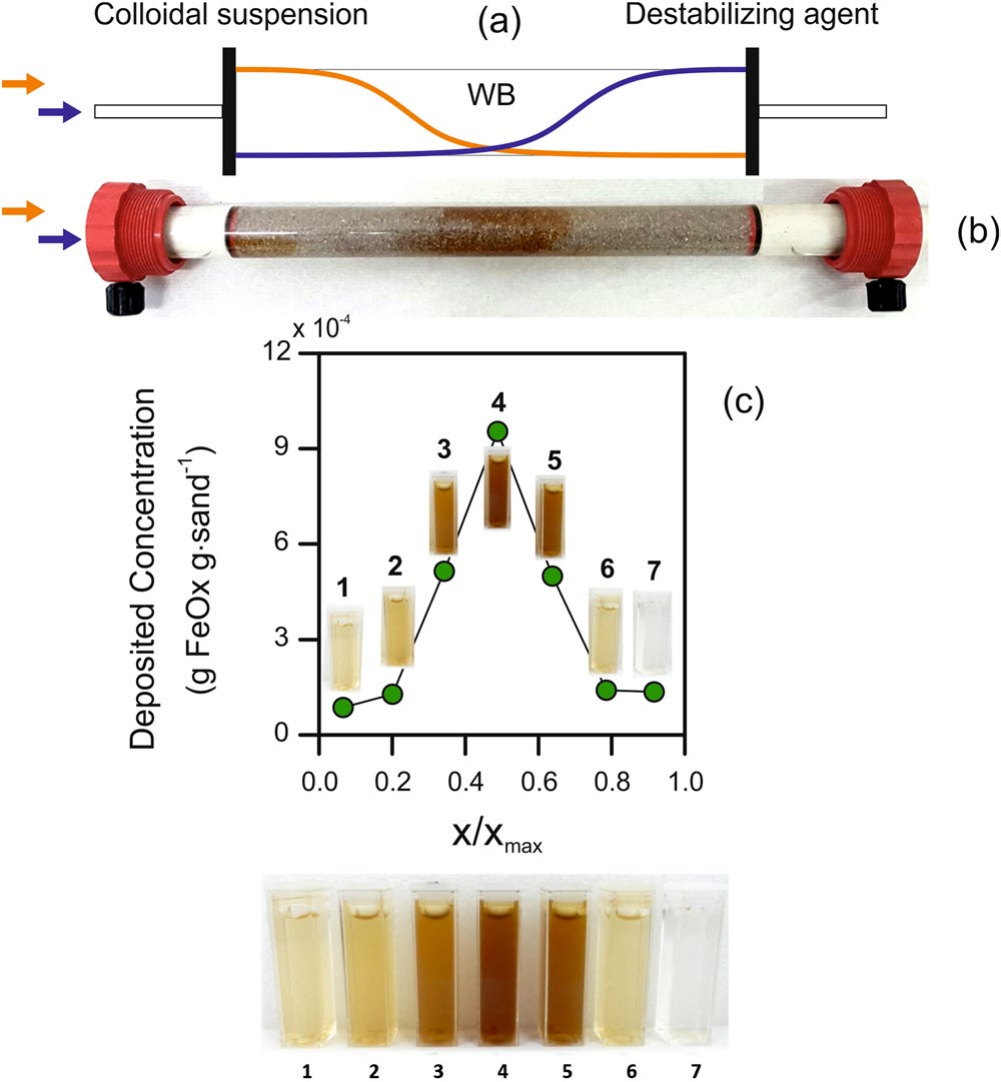
Strategy for the controlled deposition of colloidal suspensions to form reactive zones in the subsurface. The strategy is here exemplified by humic acid-coated FeOx particles used in aquifer nanoremediation. (**a**) Schematic of the deposition mechanism. Example of experimental results with deposition in the central portion of a column filled with saturated sand: (**b**) picture of the column and (**c**) concentration profile at the end of the experiment.

Employing a benign destabilizing agent is a simple and versatile method to control the fate of reactive colloidal suspensions in porous systems. In particular, tuned deposition of the particle suspension may be achieved through the controlled mixing of the slurry with the destabilizing agent in a desired portion of the medium. The destabilizing agent and the particle suspension are sequentially injected (at a single point), separated by a pulse injection of ultrapure water (acting as buffer agent (WB), which avoids premature contact between the particles and the destabilizer). The timing and location of the mixing are tuned taking advantage of the mechanisms controlling solute and particle transport in porous media, namely advection, retardation, and hydrodynamic dispersion. Advection determines how fast the nanoparticle and destabilizing agent fronts advance in the porous medium. Retardation, caused by adsorption for solutes and by attachment/detachment processes for nanoparticles, decreases the velocity of the fronts. Being dependent on the properties of the substance, retardation causes the destabilizer and the nanoparticles to move with different velocities. Hydrodynamic dispersion creates tails extending both backward and forward with respect to the propagation of the fronts; the extent of such tails increases over time and space. As a consequence, the combination of differential retardation and progressive spreading causes mixing of the two fronts within the WB pulse: particles are thus destabilized and rapidly immobilized in the porous medium (see schematic in Fig. 1a). Knowing the system properties (e.g., porous medium dispersivity, transport properties and retardation factors of the particles and destabilizing agent) and the operating conditions (namely, discharge rate and pulse duration), the location where this front mixing occurs can be precisely tuned to induce particle deposition in the desired area.

This injection strategy was applied in a 1D column filled with silica sand for the immobilization of a FeOx suspension. 1D injections were realized at the inlet of a horizontal column with flow moving from left to right (refer to Fig. 1b and to the Materials and Methods section for a detailed injection protocol). The target deposition zone was the central region of the column, and divalent cations were used as destabilizing agents. Calcium and magnesium were previously shown to interact with the carboxyl groups of the humic acid layers that coat the FeOx nanoparticles, neutralizing their charges and promoting bridging between organic chains adsorbed on approaching particles^12^. Such interactions induce colloidal destabilization above a threshold cation dose, i.e., the ratio between the concentration of cations in solution and the amount of dispersed particles. Moreover, calcium and magnesium are prone to retardation in silica sand, being subject to sorption onto the sand grain. The combination of these two mechanisms was exploited in this study to achieve rapid aggregation and deposition of the nanoparticle suspensions in the saturated media, by appropriately tuning the mixing of the divalent cations with the colloidal FeOx. Because nanoparticles move at a faster velocity (*v*_*p*_) than retarded cations (*v*_*c*_) in the medium (see section S4 of the Supporting Information), the tails of the two fronts overlapped as they moved forward. As soon as the overlapping region was characterized by a cation/particle ratio larger than the threshold dose needed for destabilization, the particles rapidly aggregated and deposited, thus accumulating in the target zone (namely the center of the medium).

At the end of the test, the colloids were locally distributed in a narrow zone of this target area, as qualitatively observed by visual inspection of the column in Fig. 1c. Furthermore, particle deposition was irreversible, as flushing of the medium with ultrapure water did not result in detectable re-mobilization or movement of the particles. This result is particularly relevant when nanoparticle remobilization must be avoided, such as in the case of FeOx particles used for heavy metal adsorption. The porous medium was then divided into seven fractions along the column, each fraction placed in alkaline solution and sonicated to promote detachment of previously deposited FeOx colloids. The resulting concentration of particles in the sonication baths presented in Fig. 1c confirmed that their distribution peaked in the central portion of the porous medium.

The proposed approach may be conceptually extended for application to field scale remediation in more realistic scenarios. In field applications, the colloid and the destabilizing agents may be injected sequentially from a single point (see Fig. S1 of the Supporting Information) or, as an alternative, simultaneously by two different wells. In this configuration, particles would be highly mobile in the vicinity of their injection well and would deposit in the area between the two wells due to the formation of a mixing zone. The high mobility of the FeOx particles, combined with the possibility of controlling their distribution and fate within the aquifer, could potentially allow the obtainment of large radii of influence during slurry injection. Moreover, this procedure may be cyclically repeated from the same wells to achieve even larger or more concentrated reactive zones.

### An analytical equation guides the design of effective injection strategies

The injection strategy discussed in the previous section involved the introduction of an ultrapure water buffer in the medium between the injection of divalent cations and that of FeOx nanoparticles, as well as during the fourth and final injection stage. Introduction of a WB pulse between the two to-be-mixed fronts was necessary to achieve customized mixing away from the injection point. Control of these steps is crucial to achieve the desired accumulation of reactive particles at the target location. An analytical expression was derived to support the design of the injection strategy and to estimate case-specific injection times for particle immobilization in different systems.

**Figure 2:**
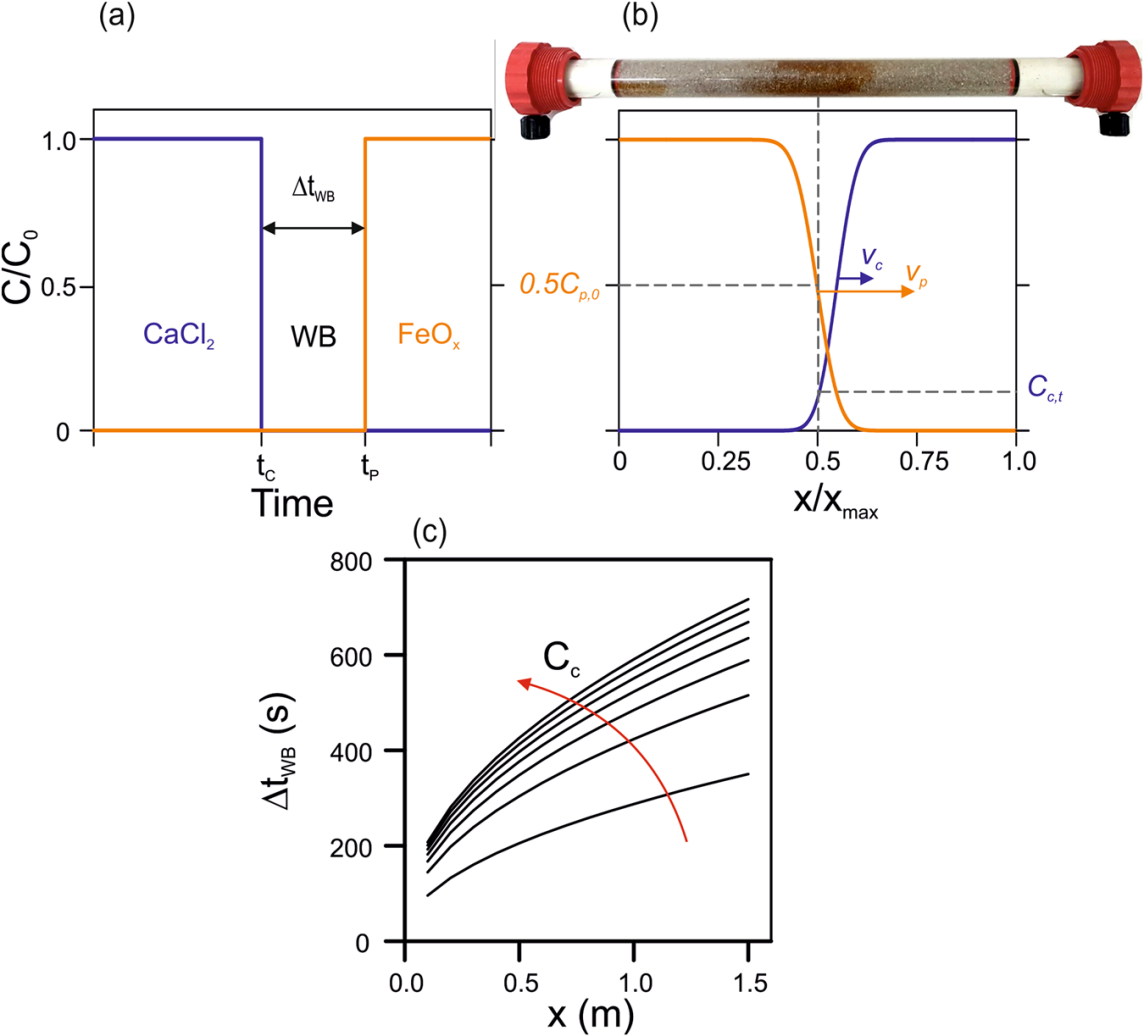
Development of the analytical expression for the design of the injection strategy for particle immobilization in the center of a saturated sandy column. (**a**) Representation of the order of injection for calcium as destabilizing agent, ultrapure water, and FeOx nanoparticles. (**b**) Condition for the immobilization of the particles in the center of the column, *x*_*t*_ = *x*_*max*_ */2*: mixing of the particle advective front and of a sufficient amount of calcium. (**c**) Duration of the required ultrapure water pulse calculated using the analytical expression as a function of the immobilization distance and the injected calcium concentration.

Figure 2a outlines the implementation steps of the immobilization protocol: a sandy column is pre-conditioned by injecting a divalent cation solution (e.g., calcium chloride) for a time, *t*_*c*_. Subsequently, a Δ*t*_*WB*_ long pulse of ultrapure water is injected, followed by a pulse of FeOx nanoparticles at time ${t}_{p}={t}_{c}+{\rm{\Delta }}{t}_{WB}$. Flow direction is kept constant (in Fig. 2, from left to right) and the inlet point is the left-hand end of the column. Mobility tests showed that goethite nanoparticles are transported faster than the destabilizing agents (*v*_*p*_ > *v*_*c*_) in the saturated porous media considered in this study, and that also dispersivities are different^24^. Values of velocities and transport parameters for the various species are reported in section S4 of the Supporting Information. The reactive zone is therefore expected to form in the portion of the column where the particles, moving faster than the divalent cations, reach the retarded destabilizing agent, also aided by hydrodynamic dispersion that stretches the moving fronts.

It may be assumed that the position, *x*_*t*_, where the advective front of the particle plume (corresponding to half of the injected particle concentration, *C*_*p,0*_) mixes with a divalent cation concentration sufficient to induce its fast destabilization (threshold concentration, *C*_*c,t*_) represents the center of the reactive zone (Fig. 2b):

1 $${x}_{t}:\{\begin{array}{c}{C}_{p}({x}_{t},t)=\frac{1}{2}{C}_{p,0}\,\\ {C}_{c}({x}_{t},t)={C}_{c,t}({C}_{p})\end{array}$$

Previous studies^12^ suggested that the threshold concentration of calcium necessary to induce fast destabilization of humic-acid stabilized FeOx nanoparticles is a linear function of the particle concentration:

2 $${C}_{c,t}=\beta \cdot {C}_{p}+\gamma $$

where *C*_*p*_ is the solid concentration of the particle suspension (ML^−3^), and *β* (NM^−1^) and *γ* (NL^−3^) are empirical coefficients derived from sedimentation experiments performed at different ionic content and particle concentrations, as described in^12^. For particles similar to those considered in this study, *β* and *γ* were found to be equal to 0.27 mmol/g and 0.5 mM, respectively. This correlation was used in this work to determine the amount of calcium or magnesium needed to destabilize the prevailing particle concentration at the advective front.

The precise duration of the WB pulse, Δ*t*_*WB*_, necessary to induce nanoparticle immobilization at a distance *x*_*t*_ from the column inlet, can be derived considering the processes controlling the transport of particles and destabilizing agent, and equation (2).

Assuming that both processes of cation adsorption and particle attachment/detachment onto the porous matrix can be approximated with a linear sorption model, the analytical equation proposed by Lapidus and Amundson^25^ (equation (S1) of the Supporting Information) can be used to describe the transport of both species. Considering that the column is preconditioned with cations and then water is injected at time *t*_*c*_, the equation describing the retarded transport of the destabilizing agent is obtained using the superposition principle applied to an approximation of the Lapidus and Amundson^25^ solution:

3 $$\frac{{C}_{c}}{{C}_{c,0}}\cong \frac{1}{2}erfc[\frac{x-{v}_{c}t}{2\sqrt{{\alpha }_{c}{v}_{c}t}}]-\frac{1}{2}erfc[\frac{x-{v}_{c}(t-{t}_{c})}{2\sqrt{{\alpha }_{c}{v}_{c}(t-{t}_{c})}}]\cong 1-\frac{1}{2}erfc[\frac{x-{v}_{c}(t-{t}_{c})}{2\sqrt{{\alpha }_{c}{v}_{c}(t-{t}_{c})}}]$$

where the first term approaches the unit when the cation front is fully developed within the porous medium.

Similarly, for nanoparticles injected at time *t*_*p*_, the transport equation is:

4 $$\frac{{C}_{p}}{{C}_{p,0}}\cong \frac{1}{2}erfc[\frac{x-{v}_{p}(t-{t}_{p})}{2\sqrt{{\alpha }_{p}{v}_{p}(t-{t}_{p})}}]$$

Assuming that the reactive zone is formed within the buffer pulse where the advective front of nanoparticles mixes with a divalent cation concentration sufficient to induce its fast destabilization, and that this threshold concentration is given by equation (2), the analytical expression for the buffer pulse duration Δ*t*_*WB*_ required to induce particle immobilization at a distance *x*_*t*_ from the column inlet can be derived coupling equations (2–4):

5 $$\begin{array}{rcl}{\rm{\Delta }}{t}_{WB} & = & {(t-{t}_{c})|}_{{C}_{c}({x}_{t},t)={C}_{c,t}}-{(t-{t}_{p})|}_{{C}_{p}({x}_{t},t)=\frac{1}{2}{C}_{p,0}}\\  & = & \frac{2{x}_{t}+{E}^{2}\cdot {\alpha }_{c}+\sqrt{{E}^{4}\cdot {\alpha }_{c}^{2}+4L\cdot {E}^{2}\cdot {\alpha }_{c}}}{2{v}_{c}}-\frac{{x}_{t}}{{v}_{p}}\end{array}$$

Here, *E* is a case-specific term depending on the particle-destabilizer interaction equal to:

6 $$E=2erf{c}^{-1}(2-\frac{2\gamma +\beta \cdot {C}_{p,0}}{{C}_{c,0}})$$

where *C*_*p,0*_ and *C*_*c,0*_ are the concentrations of particles and destabilizing agent in the injected suspensions, respectively, and *erfc* ^−1^ is the inverse of the complementary error function.

Equation (5) can be applied to any porous medium-nanoparticle-destabilizer system with known flow and transport properties. All parameters needed to implement the design equation can be determined with tailored column transport injecting, separately, a tracer, the destabilizing agent, and the particles (see details in Supporting Information).

Figure 2c illustrates the use of equation (5) for the calculation of Δ*t*_*WB*_ as a function of two important operating parameters: (i) the distance from the column inlet where particle immobilization is desired, *x*_*t*_, and (ii) the concentration of the injected destabilizing agent. If the target immobilization distance is greater, the effects of hydrodynamic dispersion and of the difference in the transport velocity facilitating particle-destabilizer contact should be counteracted by greater values of Δ*t*_*WB*_. Analogously, when high concentrations of destabilizing agent are injected into the medium, a more significant effect of dispersion is expected due to the larger concentration gradients. Therefore, greater separation distances between the two plumes are required to avoid premature deposition of the particles.

**Figure 3:**
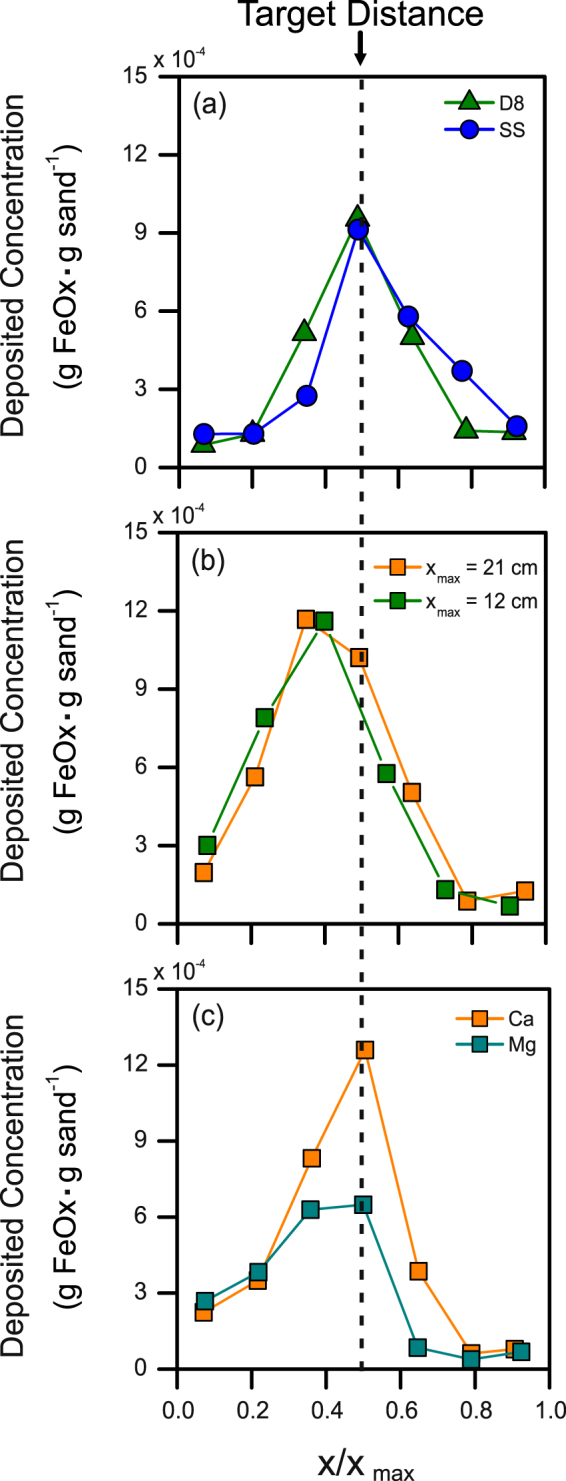
Nanoparticle accumulation in the center of sand-packed column columns. Controlled deposition of humic acid-coated FeOx nanoparticles achieved employing (**a**) two different sandy media; (**b**) two different column lengths; (**c**) two different destabilizing agents.

By applying equation (5) to achieve the desired deposition of the FeOx particles in different systems its validity was probed. Experiments were performed changing sand granulometry and column length, and by using magnesium instead of calcium as a destabilizing agent. Table 1 reports the WB injection durations predicted by equation (5) and subsequently used in the immobilization experiments. In all cases, application of the equation resulted in the successful formation of a narrow reactive zone of immobilized nanoparticles in the target area, i.e., the central portion of the column; see Fig. 3. On the contrary, shorter or longer pulses of water than those predicted by equation (5) caused premature or late particle deposition; one example of such tests is presented in Fig. S2 of the Supporting Information. Further inspection of Fig. 3, reveals that the use of magnesium as destabilizing agent resulted in a lower particle concentration in the clustering zone compared to that achieved with calcium in analogous tests. The dose of magnesium necessary to destabilize the FeOx suspension is higher than that of calcium, so a more concentrated front of divalent cations was introduced during the first injection stage. This resulted in larger dispersion and, consequently, in poorer control of mixing within the medium, as also suggested by Fig. 2. This observation suggests that, while different agents may be successfully employed to induce colloidal destabilization, more efficient compounds result in a superior accumulation of particles in the reactive zone.

**Table 1:** Summary of the immobilization experiments performed varying the type of porous medium, the type and concentration of destabilizing agent and the length of the column. Duration of the water buffer pulses predicted by the analytical equation for each column immobilization test is also reported.

Sand	Test	Destabilizing agent	Column length	*t*_*WB*_
Dorsilit n.7	A	20 mM CaCl_2_	11.4 cm	99 s
	B	20 mM CaCl_2_	21.4 cm	151 s
	C	100 mM CaCl_2_	20.1 cm	232 s
	D	100 mM MgCl_2_	21.0 cm	205 s
Dorsilit n.8	E	20 mM CaCl_2_	20.5 cm	178 s
Sibelco S1	F	20 mM CaCl_2_	21.0 cm	248 s

### Upscale of the injection strategy and implications for field application

**Figure 4:**
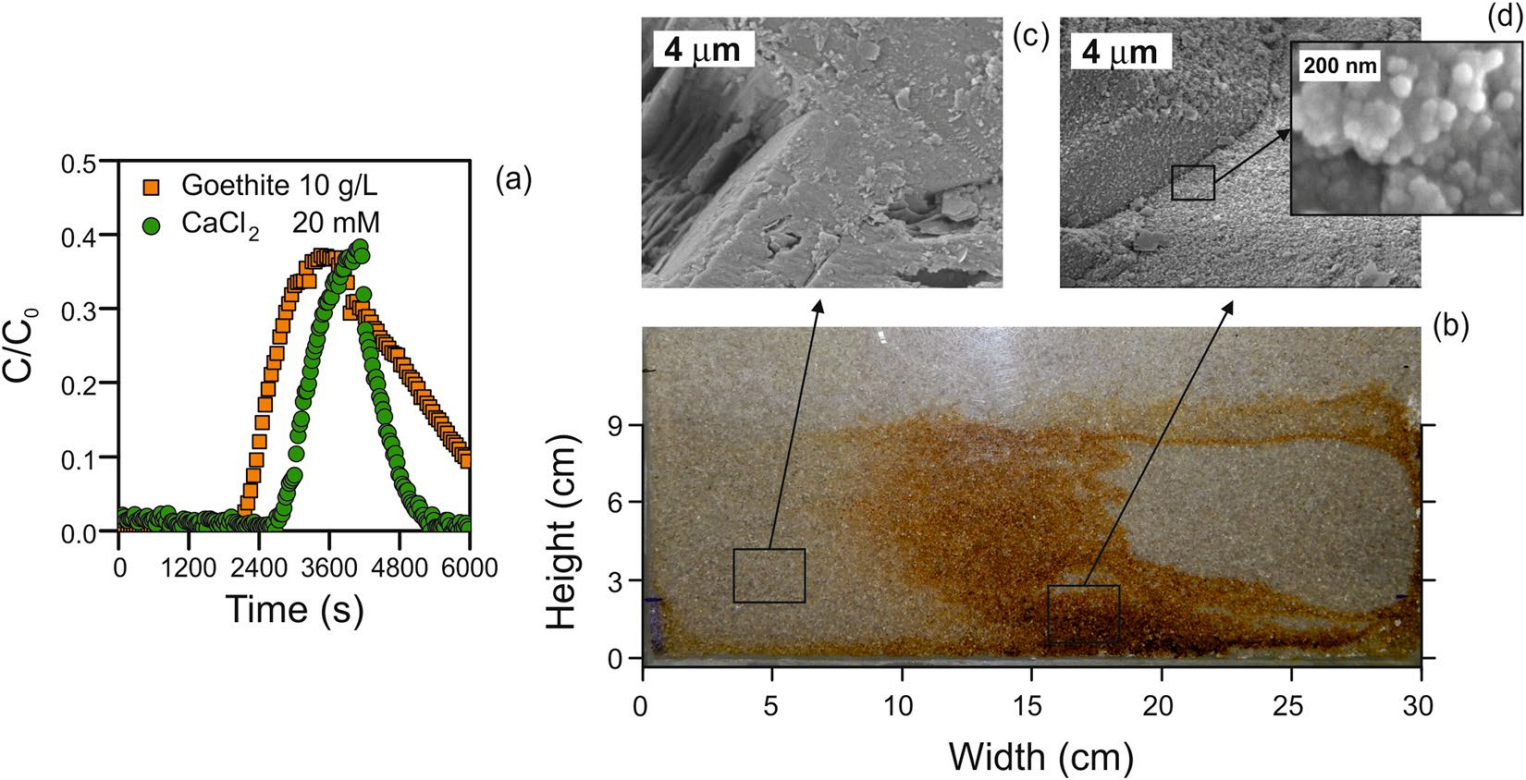
Nanoparticle deposition in a sand-packed 2D experimental setup. (**a**) Comparison of transport of calcium chloride solutions and concentrated FeOx suspensions. (**b**) Image of the 2D model after application of the proposed strategy. (**c**) Representative SEM micrographs of sand collected outside and (**d**) inside the target zone, the latter covered with nanoparticles after immobilization of nano-FeOx due to the application of the proposed strategy.

Tests were also conducted in a 2D setup filled with sand and partially saturated in the vertical dimension, with results summarized in Fig. 4. Flow was induced by using a vertical line of injection points on the left-hand boundary of the setup and a vertical line of extraction points on the right-hand boundary, simulating injection and extraction from two single wells in field application. The nano-FeOx slurries were injected at high concentration (10 g/L), similar to that necessary in field applications. Consistent with the experiments performed with 1D setups, breakthrough curves suggested that FeOx nanoparticles suspended in indifferent solutions moved faster than calcium within the medium, as the advective front reached the extraction boundary in a shorter time (see Fig. 4a). The vast majority of particles in these tests were retrieved at the outlet of the system, although full recovery was relatively slow due to backward tailing following arrival of the advective front.

The proposed strategy was applied again with the objective of making particle accumulate in the central portion of the sandy 2D system. An appropriate injection sequence of a calcium solution, water, and then the colloidal suspension was designed based on the analytical expression discussed above. The sequence caused the immobilization of a relatively concentrated amount of particles near the target zone, as visually observed in Fig. 4b. Particles were irreversibly deposited onto the sand grains as flushing of the system with ultrapure water did not result in significant re-mobilization of the colloids. Because of the high particle concentration, the density of the injected suspension is significantly higher than that of water; thus, also gravity contributed to transport by inducing particle migration towards the bottom of the artificial aquifer. Therefore, the immobilization zone was more concentrated towards this portion of the system. This result proves that gravity effects may be relevant under less idealized conditions, and must be taken into account when the proposed strategy is applied in the field. The SEM micrographs reported in Fig. 4 were obtained during visualization of the surface of sand grains collected from inside (Fig. 4d) and outside the target zone (Fig. 4c) at the end of a test. Sand grains outside the target zone showed a clean surface with only few deposited particles. Conversely, sand grains collected within the target zone displayed a large quantity of deposited iron oxide particles completely covering the minerals and forming multilayers that should prove remarkably effective in achieving high reactivity in field applications.

While this experiment was carried out in a simplified and controlled environment, this study suggests that the proposed approach may be successfully applied to control particle deposition also in more complex geometries. However, when this method is applied to optimize nanoremediation in natural aquifers, factors associated to real soil conditions, such as soil heterogeneity, natural content of destabilizing agents in groundwater, and density-driven transport may result in a different emplacement of the particles from that predicted by the model. As an example, the presence of natural destabilizing agents and the soil heterogeneity, which enhances the porous medium dispersivity and consequently the particle-destabilizer mixing, may result in an earlier deposition of the particles. Longer buffer pulses than those predicted by equation (5) may therefore be required for the particles to reach the target zone. Concentrated suspensions of nanoparticles may instead migrate downward and accumulate in the lower part of the aquifer, thus reducing the effectiveness of the remediation along the overall depth of the contamination. In this case, the immobilization strategy may be customized to mitigate this undesired effect, e.g. a lower particle concentration may be applied and the injection procedure cycled until a sufficient amount of particles is delivered within the reactive zone. A careful and case-specific design of the injection strategy is therefore necessary to achieve a targeted deposition of the reactive nanoparticles in real systems. In this sense, 3D numerical codes^26^ may be useful tools to predict nanoparticle transport and their interaction with the destabilizing agent in such complex scenarios.

## Conclusions

In this work, an innovative single point injection strategy was proposed and applied to control the fate of humic acid coated goethite nanoparticles, an effective reagent for the remediation of aquifer systems contaminated by heavy metals. The proposed strategy allowed the creation of reactive zones in the center of 1D and 2D experimental setups with a single injection point or well. The controlled deposition of nanoparticles at the desired location was achieved by tuned sequential injection of pulses of a particle suspension, a solution of divalent cations (used as destabilizing agent), and water (used to separate the two interacting fluids). The design of the immobilization experiments and, in particular, the estimation of the duration of the various injection phases, was guided by a simple analytical expression derived from first principles of advective-dispersive transport. This equation was validated by successful tuning experiments in which iron oxide nanoparticles were immobilized in sandy columns under different operating conditions.

This method may be directly used to optimize the design of field scale iron oxide-based nanoremediation, and thus achieve particle accumulation in a narrow target zone and reduction of the amount of lost reactive material. Large radii of influence may be obtained by repeating the injection procedure and by applying different operating conditions (e.g., different flow rates or length of the water buffer step) at every cycle, thus creating wide reactive zones with only a single injection well (therefore reducing the total number of injection wells). Finally, this method guarantees irreversible deposition of the FeOx in the porous medium, thus minimizing the possible remobilization of particles that may otherwise act as potential vehicle for heavy metals adsorbed on their surface.

The proposed injection strategy was exemplified for the specific oxide-divalent cation interaction: FeOx stabilized with humic acids undergo fast aggregation and sedimentation when a threshold dose of divalent cations is reached. However, the strategy may be easily generalized and extended for application to any other colloidal suspensions, provided that a suitable destabilizing agent is first identified. As such, the approach proposed in this work represents an important step forward in the field of nanoremediation, since for the first time it was possible to achieve control on the short- and long-term distribution of engineered particles upon injection. The predictive equation developed in this study is flexible and easy to apply, and actually represents the heart of the remediation strategy.

## Materials and Methods

### Solutions and suspensions

A stock suspension of colloidal goethite nanoparticles (concentration 110 g/L) was provided by University of Duisburg-Essen (Germany), where it was produced according to US patent 8,921,091 B2^11^. The nanoparticles were coated with humic acids to ensure colloidal stability at high solid concentrations, as demonstrated by previous studies^14^. All transport tests were performed at a solid concentration of 10 g/L, obtained by dilution of the stock suspension with deionized (DI) water, with no addition of buffer compounds. The pH of the diluted particle suspension was always approximately 8. CaCl_2_ (10, 20, or 100 mM) and MgCl_2_ (100 mM) solutions were used as destabilizing agent for the immobilization tests. NaCl (50 mM) and bromophenol blue (100 mg/L) were used as tracers for characterization of the porous media. All the solutions were degassed in a vacuum environment prior to use.

### Porous media

Three types of quartz sand were employed to pack columns and 2D systems, namely, Dorsilit n.7 (Dorfner, Germany), Dorsilit n.8 (Dorfner, Germany), and Sibelco S1 (Sibelco Italia S.p.A., Italy). Dorsilit n.7 and Dorsilit n.8 are high purity sands, composed of quartz (98.9%) and a minor percentage of K-feldspar as microcline (average density of sand: 2.64 g/cm^3^), with rounded grains with nominal size ranges of 0.6–1.2 mm and 0.3–0.8 mm, respectively. Sibelco S1 is a natural quartz sand with a measured grain size of d_10_ = 0.65 mm, d_50_ = 0.95 mm, and d_90_ = 1.45 mm. Prior to use, the sand was thoroughly cleaned by sonication in sequential baths of NaOH 10 mM and DI water (the cycle was repeated twice), then placed in DI water under vacuum conditions to completely hydrate the grains.

### Immobilization experiments

#### 1D column setups

1D experiments were performed in a custom-made Plexiglass column with an inner diameter of 16 mm. This column was wet packed with clean sand to a total length of around 20.8 ± 0.5 cm, unless otherwise stated. The particle suspension and the salt solutions were injected into the porous medium with a peristaltic pump (Ismatec ms-4/06 Reglo) at constant flow rate of 1.2 × 10^−8^ m^3^/s. Since goethite particles are denser than water, the column was oriented horizontally to minimize the influence of sedimentation, and better approximate particle transport under field conditions^27,28^. The concentration of solutes and particles at both the column inlet and outlet was monitored online via optical density measurements using a UV-vis spectrophotometer (Specord S600, Analytik Jena, Germany) equipped with flow-through cells characterized by a 2 mm light path (Hellma, Germany). Monitoring wavelengths of 198.5 nm and 800 nm were chosen for solutes and for colloidal suspensions, respectively, having shown a linear relationship between absorbance and concentration.

Results of tracer tests performed with NaCl showed that the porosity was 0.44 ± 0.08 for porous beds packed with Dorsilit n.7, and 0.46 ± 0.05 for those packed with Dorsilit n.8 and Sibelco S1. The dispersivity and retardation factor of nanoparticles and destabilizing agents in the different porous media were also fully characterized and are reported in Table S1 of the Supporting Information. All these parameters were obtained by inverse fitting of the transport data using the software MNMs 2016 (Micro-and Nanoparticle transport, filtration and clogging Model - Suite)^26^ and used in equation (5) to design immobilization experiments.

Immobilization tests were conducted varying the type of sand, the column length, and the destabilizing agent type and concentration, in order to demonstrate the wide applicability of the strategy developed here. A total of six different systems were considered (Table 1): four systems containing Dorsilit n.7 sand, packed at two different column lengths (21 cm and 11 cm) and in the presence of three different destabilizing agents (20 mM or 100 mM CaCl_2_, or 100 mM MgCl_2_), one system with Dorsilit n.8 sand and one with Sibelco S1 sand.

The injection strategy proposed in this study involved a number of sequential steps. To begin the process, the porous medium was equilibrated with ultrapure water, followed by preconditioning through injection of few pore volumes of the desired destabilizing agent. A pulse of ultrapure water was then introduced to avoid direct contact between the destabilizing agent and the particles to be immobilized at the column inlet, and to induce the deposition of the particles in the target location, which in this study was arbitrarily chosen to be the central portion of the packed column. The subsequent step comprised injection of a pulse of the reactive goethite suspension at 10 g/L. The duration of the intermediate pulse of ultrapure water was calibrated for each test (Table 1), according to the specific operating conditions, with the support of equation (5). Subsequently, water was injected again to promote particle migration to the target zone. Finally, the column was flushed with water at high flow rate (6.24 × 10^−8^ m^3^/s) to detect any remobilization of colloids deposited during the procedure.

At the end of each immobilization test, the amount and distribution of particle concentration irreversibly retained on the surface of the sand grains was determined by dissecting the column along its length in seven parts. Alkaline extraction by addition of 10 mL of NaOH 10 mM was performed to promote particle detachment from the sand samples. Disposable plastic cuvettes having 10 mm optical path length were filled with 4 mL of the supernatant liquid obtained in the previous step. The amount of deposited particles was determined by optical density measurements at a wavelength of 800 nm.

#### 2D model

A laboratory-scale Plexiglass transparent sandbox was used to test the proposed strategy in 2D geometry. The box had dimensions of 30 × 13 × 1.2 cm (L × H × W). An injection and an extraction well were placed at each end of the domain. The same flow rate of 6.24 × 10^−8^ m^3^/s was injected (left-hand well) and extracted (right-hand well) through a multi-channel peristaltic pump (Ismatec ms-4/08 Reglo) to induce a uniform Darcy velocity of 0.35 cm/min. The sandbox was wet-packed with Dorsilit n.7 sand to mimic a homogeneous unconfined aquifer: 9 cm saturated depth topped with 3 cm unsaturated sand. A gravel layer, around 1 cm thick, was placed on top of the aquifer model to reduce capillary effects.

After packing, the sandbox was equilibrated by flushing ultrapure water for at least 3 hours before starting the transport and immobilization tests. The concentration of calcium, goethite, or bromophenol blue was continuously monitored at the domain outlet via optical density measurements using an analogous procedure to that described above. A wavelength of 590 nm was employed to analyze the concentration of bromophenol blue. The migration of the particle and dye plumes, as well as the front shapes, was visually analyzed through the transparent wall.

The test consisted of the following stages. First, a tracer test with bromophenol blue was conducted to verify the absence of preferential flow paths. Then, two transport tests were performed to characterize the individual transport of goethite nanoparticles (10 g/L) and calcium chloride (100 mM). The arrival time of the advective fronts was derived from each breakthrough curve to calibrate the length of the pulse of ultrapure water for the application of the particle immobilization protocol. Finally, immobilization was induced in the center of the sandbox with the same approach as the 1D tests: equilibration with ultrapure water, preconditioning with 50 mM CaCl_2_ solution, pulse of ultrapure water, injection of particles, and final flushing with water. Samples of sand were collected from different areas of the packed medium at the end of the experiment. The morphology of the sand surface was analyzed through field emission scanning electron microscopy (FESEM) to evaluate the grain coverage by particles after their immobilization. The instrument was the MERLIN model by ZEISS, equipped with state-of-the-art GEMINILIS column ensuring accurate control of spot and current. The samples collected within the target zone were sputter-coated with a layer of chromium (8.0 nm) to prevent charging.

### Data Availability

The datasets generated during and analyzed during the current study are available from the corresponding author on reasonable request.

## Electronic supplementary material

Supporting Information
