# Supplementary material for: Controlled Deposition of Particles in Porous Media for Effective Aquifer Nanoremediation
Source: Sci Rep. 2017 Oct 11;7:12992. doi: 10.1038/s41598-017-13423-y (PMC5636825; doi:10.1038/s41598-017-13423-y)
Supplement: Supplementary file 1 — Supporting Information [file 41598_2017_13423_MOESM1_ESM.pdf]

SUPPORTING INFORMATION

Controlled Deposition of Particles in Porous Media for Effective Aquifer Nanoremediation

Carlo Bianco, Janis Eneida Patiño Higuita, Tiziana Tosco, Alberto Tiraferri, Rajandrea Sethi

Department of Environment, Land and Infrastructure Engineering (DIATI)

Politecnico di Torino

Corso Duca degli Abruzzi 24, 10129 Torino, Italy

This Supporting Information file is 7 pages long and it contains 3 figures and 1 table.

Table of Contents

S1. Possible approach of the injection strategy for field application........ 2

S2. Analytical solution for one-dimensional transport in porous media 3

S3. Particle immobilization before the center of the column. 4

S4. Determination of transport parameters 5

S1. Possible approach of the injection strategy for field application.

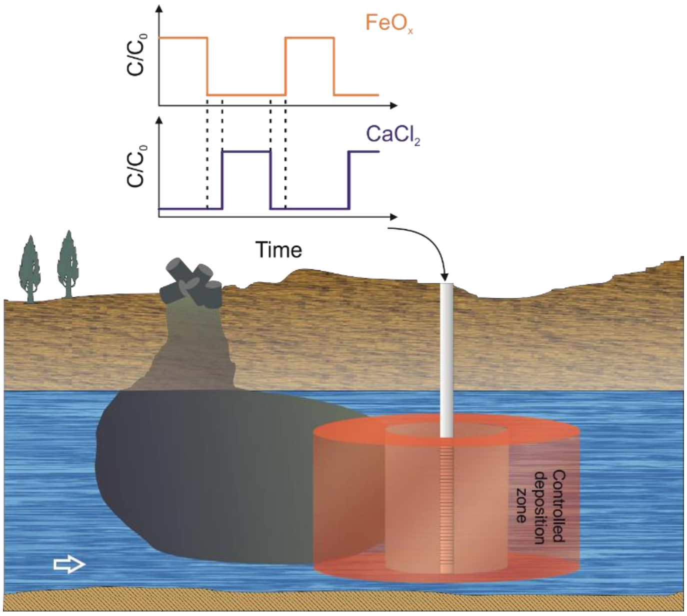

Figure S1. Approach for the field application of the injection strategy proposed for the optimization of nanoremediation by the in-situ immobilization of nanoparticles. The stable colloidal suspension of engineered nanoparticles and the destabilizing agent are sequentially injected from the same injection well, separated by a water buffer pulse. The nanoparticles deposit in the area where the front of nanoparticles mixes with the destabilizing agent plume, creating a reactive zone for the pollutant degradation.

S2. Analytical solution for one-dimensional transport in porous media

The one dimensional advective-dispersive transport of a generic species subject to linear sorption in a semi-infinite porous medium in the presence of a uniform flow field is described by the following analytical solution [1,2]:

$$\frac{C}{C_0} = \frac{1}{2}\left\{erfc\left[\frac{x - v_r t}{2\sqrt{\alpha_r v_r t}}\right] + \exp\left(\frac{x}{\alpha_r}\right) erfc\left[\frac{x + v_r t}{2\sqrt{\alpha_r v_r t}}\right]\right\} \cong \frac{1}{2} erfc\left[\frac{x - v_r t}{2\sqrt{\alpha_r v_r t}}\right] \qquad \text{(S1)}$$

where x and t are the space and time coordinates, respectively, v_r (L T^{-1}) is the species retarded velocity, lower than the pore water velocity, v, and α_r is the dispersivity coefficient (L).

S3. Particle immobilization before the center of the column.

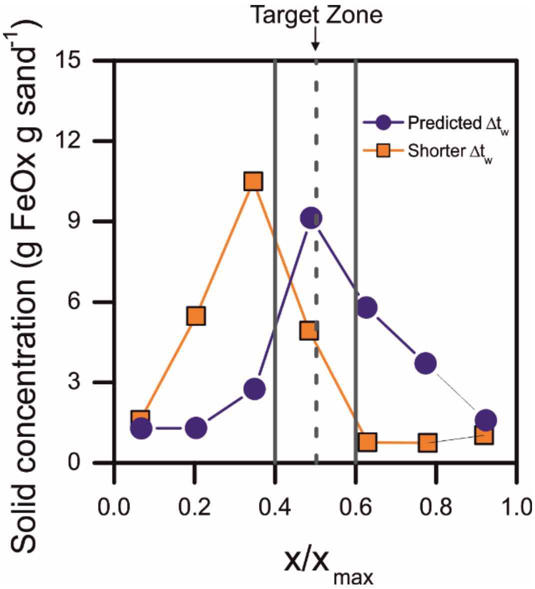

Figure S2. Immobilization tests performed in 21 cm long columns packed with Sibelco S1 sand using 20 mM $CaCl_2$ solutions as destabilizing agent. The tests were performed using two different durations of the water pulse between particle and divalent cation injections. The first test (blue circles) used the t_{WB} = 248 s as predicted from eq. 3 in the main text for x_t equal to half column (x_{max} = 10.5 cm). A second experiment was then performed applying a shorter water pulse (200 s) (orange squares). The use of a shorter water pulse resulted in the formation of the reactive zone closer to column inlet and outside of the target zone, because of the earlier contact between particles and divalent cations.

S4. Determination of transport parameters

Transport parameters needed to implement eq. 3-4 (main text) can be determined from column transport tests and colloidal stability tests. In particular, the parameter E is obtained from colloidal stability studies similar to those reported in Tiraferri, et al. [3]. The parameters α_c, v_c and v_p are obtained via least-square fitting of experimental breakthrough curves obtained from column transport tests of tracer, particles, and calcium chloride. Assuming that sorption follows a linear isotherm, eq. S1 can be used to fit the experimental data. In this case, the velocity of the retarded species can be written as:

$$v_r = \frac{q}{\varepsilon R} \qquad \text{eq. S2}$$

where q is the specific discharge (i.e. discharge rate divided by the cross section of the column), ε is the effective porosity available for flow, and R is the retardation factor, equal to 1 for a tracer, and higher than 1 for retarded substances. The effective porosity ε is independent of the transported species, while R and α_r, as a general rule, are not [4].

Experimental breakthrough curves can be least-squares fitted to eq. S1 to determine parameters of eq. 3 in the main text following this approach (in this work, the software MNMs, http://areeweb.polito.it/ricerca/groundwater/software/MNMs.php, was used to this aim):

- A tracer test (i.e. injection of a tracer solution - in this work calcium chloride - in the column previously saturated with tracer-free solution) is run and the breakthrough curve is fitted to eq. S1 assuming $R=1$. ε and α_t are thus determined.
- A solution of the destabilizing agent (e.g., calcium chloride) is injected into the column previously saturated with calcium-free solution. The breakthrough curve is fitted to eq. S1 using ε from the tracer test: α_c and R_c are thus determined, and v_c is calculated using eq. S1.
- The nanoparticle suspension is injected into the column previously saturated with particle-free solution. The breakthrough curve is fitted to eq. S1 using ε from the tracer test: α_p and R_p are thus determined, and v_p is calculated using eq. S2.

An example of experimental and modeled breakthrough curves for calcium chloride and FeOx nanoparticles in Sibelco sand is reported in Figure S3. Fitted parameters for the experimental conditions explored in this work are reported in Table S1.

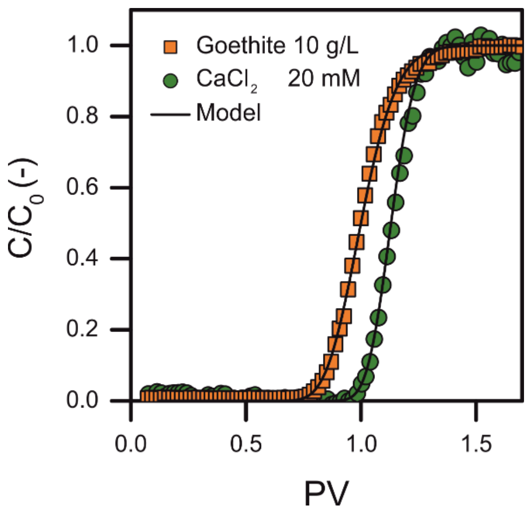

Figure S3. Comparison of nanoparticle and calcium arrival time. Breakthrough curves of humic acid-coated goethite nanoparticles at 10 g/L solid content in DI water (orange square), of a 20 mM solution of $CaCl_2$ (green circles) are shown. Modeled breakthrough curves obtained by iverse fitting of the experimental data using the MNMs software are reported as black lines. In the y-axis, the value of concentration at the outlet with respect to the injected concentration, C_0, calculated from values of light absorbance at 800 nm wavelength (particles) and 198.5 nm (calcium) based on calibration lines. In the x-axis, the pore volumes injected into the column. The fluids were introduced at time 0. The key experimental conditions were: pH 7.5-8, pore volume 20.2 mL, temperature 25 °C, and Darcy's velocity 7.8×10^{-5} m/s.

Table S1. Dispersivity, retardation coefficient and transport velocity of calcium, magnesium and FeOx nanoparticles in different porous media. Data were estimated using the software MNMs by inverse fitting of individual transport tests.

Sand	Parameter	Calcium	Magnesium	FeOx
Dorsilit n.7	Dispersivity	9.7×10^{-4} m	9.1×10^{-4} m	1.4×10^{-3} m
	Retardation coefficient	1.05	1	1
	Effective velocity	1.68×10^{-4} m/s	1.76×10^{-4} m/s	1.76×10^{-4} m/s
Dorsilit n.8	Dispersivity	8.7×10^{-4} m	-	1.1×10^{-3} m
	Retardation coefficient	1.03	-	1
	Effective velocity	1.26×10^{-4} m/s		1.3×10^{-4} m/s
Sibelco S1	Dispersivity	9.0×10^{-4} m	-	2.0×10^{-3} m
	Retardation coefficient	1.1	-	1
	Effective velocity	1.29×10^{-4} m/s		1.46×10^{-4} m/s

References

1 Lapidus, L. & Amundson, N. R. Mathematics of Adsorption in Beds. VI. The Effect of Longitudinal Diffusion in Ion Exchange and Chromatographic Columns. *The Journal of Physical Chemistry* **56**, 984-988, doi:10.1021/j150500a014 (1952).

2 Ogata, A. & Banks, R. B. A solution of the differential equation of longitudinal dispersion in porous media. Report No. 411A, (1961).

3 Tiraferri, A., Saldarriaga Hernandez, L. A., Bianco, C., Tosco, T. & Sethi, R. Colloidal behavior of goethite nanoparticles modified with humic acid and implications for aquifer reclamation. *Journal of Nanoparticle Research* **19**, 107, doi:10.1007/s11051-017-3814-x (2017).

4 Chrysikopoulos, C. V. & Katzourakis, V. E. Colloid particle size-dependent dispersivity. *Water Resources Research* **51**, 4668-4683, doi:10.1002/2014wr016094 (2015).
